# Sessile drops in weightlessness: an ideal playground for challenging Young’s equation

**DOI:** 10.1038/s41526-021-00153-9

**Published:** 2021-08-04

**Authors:** Marc Medale, David Brutin

**Affiliations:** Aix Marseille Université, CNRS, IUSTI, Marseille, France

**Keywords:** Engineering, Aerospace engineering

## Abstract

Sessile drop creation in weightlessness is critical for designing scientific instruments for space applications and for manipulating organic or biological liquids, such as whole human blood or DNA drops. It requires perfect control of injection, spreading, and wetting; however, the simple act of creating a drop on a substrate is more complex than it appears. A new macroscopic model is derived to better understand this related behavior. We find that, for a given set of substrate, liquid, and surrounding gas properties, when the ratio of surface free energies to contact line free energy is on the macroscopic scale, the macroscopic contact angle can vary at static equilibrium over a broad volume range. It can increase or decrease against volume depending on the sign of this ratio up to an asymptotic value. Consequently, our model aims to explore configurations that challenge the faithful representativity of the classical Young’s equation and extends the present understanding of wetting.

## Introduction

**Fig. 1:**
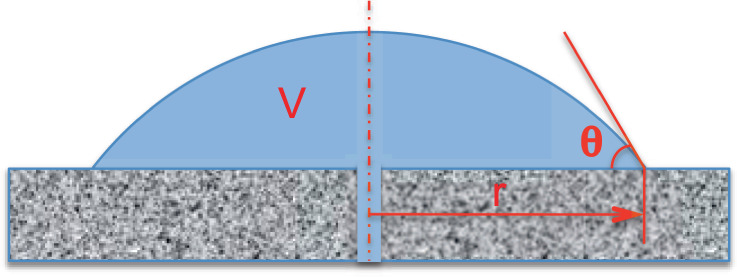
Sketch of sessile drop, injected from below through a sub-millimetric hole in the substrate. V is drop volume, r is wetted radius, $\theta$ is macroscopic contact angle.

A sessile drop is a liquid drop deposited on a solid substrate and surrounded by a gaseous environment. Sessile drops are ubiquitous all around us, either in natural environment (raindrops on a surface), or in industrial processes in which liquids intervene. For scientific purposes, they can be processed by injecting liquid through a small hole in the substrate, as shown in Figure 1.

The underlying scientific question is, given a set of solid–liquid-gas material properties and injected volume, what is the macroscopic size and shape of the resulting sessile drop?

The first answer to this question is provided by the classical Young’s equation^1^, Eq. (1), which was formulated roughly two centuries ago. Nevertheless, it remains an undisputed reference for the determination of macroscopic contact angles, denoted by *θ* (cf. Fig. 1), according to its famous relationship:

1 $${\sigma }_{sg}-{\sigma }_{sl}={\sigma }_{lg}\ cos\theta$$

where *σ*_*sg*_, *σ*_*sl*_, and *σ*_*lg*_ are the interface free surface energies, and the subscripts *s*, *l*, and *g* represent a solid, liquid, and gas, respectively. The effectiveness of Young’s equation lies in its ability to translate subtle and complex molecular-scale physico-chemical interactions near material interfaces to the macroscopic scale in a simple manner. However, as Young’s equation is assumed to be general, it remains unclear why neither the contact line free energy nor gravity forms a part of the macroscopic contact angle relationship in this equation. One possible answer is that in most classical cases, these two parameters are negligible with respect to the surface free energies, inducing only second-order deviations as compared with the leading mechanisms. However, from a scientific standpoint, the question is whether there are configurations for which these two contributions could lead to non-negligible effects.

Indeed, several experiments have shown some influence of volume on the macroscopic contact angle under Earth’s gravitational conditions^2–7^. These experiments show that a volume dependence exists for macroscopic sessile drops on Earth for several sets of liquid/substrate/surrounding gas (water on PTFE in air, alkanes on Teflon in air, etc.), provided that the sessile drop size remains below its capillary length. Above the latter, self-adaption of the local interface curvature to the hydrostatic pressure is the dominant contact line mechanism. Hence, the static macroscopic contact angle becomes independent of the drop volume. To explain the influence of volume on macroscopic contact angle, Boruvka and Neumann^8^ introduced the concept of line tension associated with the triple line (first introduced by Gibbs more than a century and a half ago) and suggested a modified version of Young’s equation to account for it^4,6,7^. However, no definitive consensus has emerged regarding a physical understanding of the translation of events from the microscopic to the macroscopic scale^9,10^, but a recent and sounding review provides many inputs that enable to make its own view on this tentacular line tension topic^11^.

Thus, revisiting a way to determine the macroscopic contact angle of sessile drops in a manner different from the well-established one could either be tremendously risky at the worst or very ambitious at best. However, this is the goal of the present study in order to explore configurations that challenge the faithful representativity of the classical Young’s equation.

Indeed, a second possibility or alternate approach is to determine the macroscopic contact angle of sessile drops following the pioneering work of Laplace^12^, who, unlike Young, considered a macroscopic mechanistic approach. This resulted in the famous Young–Laplace equation that governs the shape of any interface separating non-miscible fluids, as it relates the capillary pressure to the liquid–gas surface tension and mean interface curvature. Unfortunately, this governing equation is a highly nonlinear differential equation that has no analytical solution in its most general form. However, in the case of axisymmetric sessile drops, Bashforth and Adams^13^ were the first to provide very accurate numerical solutions by means of high-order Taylor series expansions, which agreed well with the best experimental results available at that time. Based on this pioneering numerical work, numerous improvements have been achieved to enhance the capabilities and accuracy of the original calculation methods^14^. Notably, minimizing the total free energy of a sessile drop enables the recovery of both the Young–Laplace equation and Young’s equation for the contact angle in completely independent ways^15^. However, most existing numerical methods for axisymmetric sessile drops^16–19^ assume knowledge of at least two geometrical quantities from among sessile drop volume, height, or wetted radius. Only when this information is available can these methods be used to determine the static contact angle for a given set of material properties or to deduce some of the latter when all geometrical quantities are known through experiments.

To the best of the authors’ knowledge, no such influence of volume on macroscopic contact angle has been reported yet in weightlessness. Therefore, the present study focuses on the potential influence of contact line tension on macroscopic contact angle and on providing a better design for future experiments on weightless sessile drops. Therefore, geometrical data on sessile drops are not assumed to be a prerequisite and one seeks for the complementary approach with respect to classical one. Furthermore, it is assumed that the set of material properties is known, along with one of the geometrical target quantities of the sessile drop, either its volume, height, or wetted radius. Then, all other related geometrical quantities to be determined (static macroscopic contact angle, etc.) are predicted. In addition, some more generic, albeit related questions are addressed: What are the equilibrium sizes and macroscopic shape of a weightless sessile drop of a prescribed volume for a given set of properties of liquid, substrate, and surrounding gas materials? How do the related geometrical quantities, such as static macroscopic contact angle, drop wetted radius, and height evolve with respect to sessile drop volume? How does the bulk pressure in the sessile drop evolve in turn?

## Results

### Exploring the parameter space

As the proposed model is proven to be capable of reproducing the volume dependence of the macroscopic contact angle (see the “Methods” section for all notations), the parameter space in weightlessness is explored. For conciseness, the focus is on the main feature of the model through parametric studies that investigate the influence of the three leading parameters, i.e., sessile drop volume ${{{\mathscr{V}}}}$, surface free energy ratio ${{{\mathscr{S}}}}$, and the sign of the contact line tension *σ*_*slg*_. These parametric studies were performed by solving Eqs. (7) for the macroscopic contact angle in the range $\left]0,\pi \right[$ using the Mathematica software^20^. Once the macroscopic contact angle is computed, the three related geometrical quantities of interest—drop wetted radius, height, and sphere radius—can be explicitly obtained using Eq. (6A)(6B)(6C)(a–c).

#### Influence of volume for given sets of liquid–solid–gas properties

**Fig. 2:**
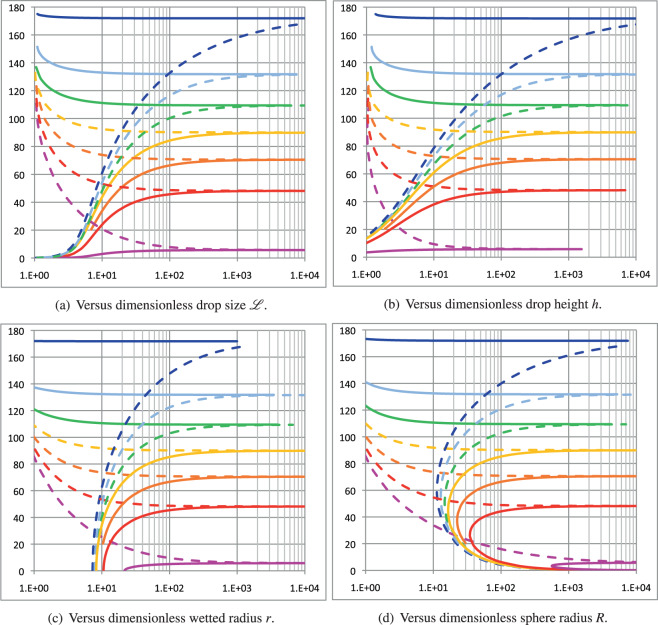
Macroscopic contact angle versus dimensionless geometrical quantities (${{{\mathscr{L}}}}$, *h*, *r* and *R*), for ${{{\mathscr{S}}}}=-0.99$ (blue), −2/3 (cyan), −1/3 (green), 0 (yellow), 1/3 (orange), 2/3 (red) and 0.99 (magenta). **a** versus dimensionless drop size ${\mathcal{L}}$; (**b**) versus dimensionless drop height *h*; (**c**) versus dimensionless wetted radius *r*; (**d**) rersus dimensionless sphere radius *R*.

Let us first consider the influence of the sessile drop volume on its shape for various values of ${{{\mathscr{S}}}}$ and both signs of contact line tension *σ*_*slg*_. The resulting macroscopic contact angle is plotted against all dimensionless geometrical quantities (${{{\mathscr{L}}}}$, *h*, *r*, and *R*) in Figure 2, for ${{{\mathscr{S}}}}=0,\ \pm 1/3,\ \pm 2/3,\ \pm 0.99$, cf. figure caption, and ${l}_{\sigma }=\pm 1{0}^{-3}{{{\mathscr{S}}}}$, except for the case of ${{{\mathscr{S}}}}=0$, where *l*_*σ*_ is set to ± 10^−3^. Solid (dashed) lines represent the positive (negative) values of the ${{{\mathscr{S}}}}/{l}_{\sigma }$ ratio. First, the limit angle associated with this macroscopic model, denoted as *θ*_*lim*_ in Eq. (9), always tends to $\frac{\pi }{2}[1-\,{{\mbox{sign}}}\,({\sigma }_{slg})]$, regardless of the sign of ${{{\mathscr{S}}}}$. This suggests that when the length *l*_*σ*_ is commensurable at the macroscopic scale, it dictates the limit behavior when the drop size reaches its lowest macroscopic limit. Moving slightly away from singularity, the macroscopic contact angle is now well-defined and continuously increases (decreases) with the sessile drop size, depending on whether *σ*_*slg*_ is positive (negative), up to its asymptotic limit, ${\theta }_{\infty }=\arccos {{{\mathscr{S}}}}$. Thus, a crossover between hydrophobic (non-wetting) and hydrophilic (wetting) behaviors or vice-versa only occurs if the ${{{\mathscr{S}}}}/{l}_{\sigma }$ ratio is negative. Furthermore, the asymmetry with respect to *π*/2 of the trigonometric functions in Eq. (7) induces a strong asymmetry in the macroscopic contact angle for low drop sizes. The macroscopic contact angle varies monotonically with the dimensionless drop size ${{{\mathscr{L}}}}$, height *h*, and wetted radius *r*, as shown in Fig. 2a–c. However, it exhibits a non-monotonic behavior with respect to the sphere radius for positive *l*_*σ*_ (see Fig. 2d. Finally, as can be observed from Fig. 2, the chosen definition of the reference length is optimal. It enables the depiction of the full picture in the smallest span range of the drop size, as compared with other dimensionless quantities, *h*, *r*, or *R*, which would require at least one more decade. Furthermore, one can notice that the widely used plot of macroscopic contact angle versus wetted radius, Figure 2(c), does not contain the lowest asymptotic limits fairly reproduced in Fig. 2a. On the other hand, Fig. 2d interestingly indicates that positive *l*_*σ*_ always leads to the existence of a minimal sphere radius, whose value depends on ${{{\mathscr{S}}}}$. Above this minimum, any given sphere radius admits two macroscopic contact angles, as the relationship between these two related quantities is multi-valued.

**Fig. 3:**
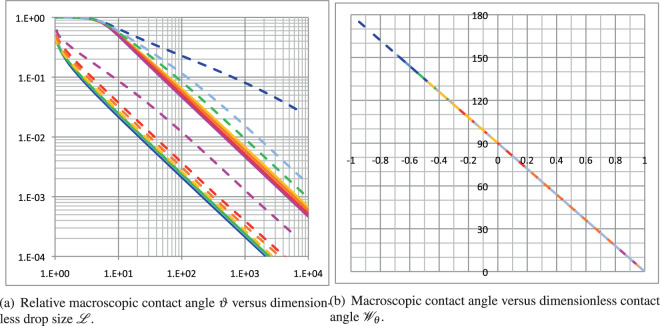
Macroscopic contact angle versus dimensionless drop size ${\mathcal{L}}$ and dimensionless number characterizing the contact angle ${\mathcal{W}_{\theta}}$. **a** Relative macroscopic contact angle ${\mathcal{\vartheta}}$ versus dimensionless drop size ${\mathcal{L}}$; (**b**) Macroscopic contact angle versus dimensionless contact angle ${\mathcal{W}}_{\theta}$.${\mathcal{W}}_{\theta}=-0.99$ (blue), −2/3 (cyan), −1/3 (green), 0 (yellow), 1/3 (orange), 2/3 (red) and 0.99 (magenta). The solid (dashed) lines represent the plots for positive (negative) ${\mathcal{S}/l_{\sigma}}$.

The definition of ${{{{\mathscr{W}}}}}_{\theta }$ is derived from the closely related behavior of the relative variation in the macroscopic contact angle over its span range, defined as ${{{\mathcal{\vartheta }}}}=\frac{\theta -{\theta }_{\infty }}{{\theta }_{lim}-{\theta }_{\infty }}$. The latter is plotted against the dimensionless drop size in Fig. 3a and emphasizes the asymmetry between the positive and negative values of the ${{{\mathscr{S}}}}/{l}_{\sigma }$ ratio. Furthermore, this log–log scale plot clearly indicates that there are three distinctive regions with specific behaviors. The first is at the upper-left corner of the plot, where curves asymptote either the ordinate axis for negative *l*_*σ*_ or the unity horizontal line for positive one. Therefore, it is a connecting region to *θ*_*lim*_, which spreads over very different drop size ranges, depending on *θ*_*lim*_. In the opposite plot corner, in the region of the largest drop sizes, the final asymptotic $1/{{{\mathscr{L}}}}$ behavior occurs. Between these two extreme regions lies the transition region, whose extension strongly depends on the ${{{\mathscr{S}}}}/{l}_{\sigma }$ ratio. These specific behaviors enable us to derive related functions, *f*_*co*_, *f*_*pa*_, defined in Eq. (10C)–(10D), which are at the core of the proposed definition of ${{{{\mathscr{W}}}}}_{\theta }$. To appreciate how representative is the proposed model, the computed macroscopic contact angle *θ* is plotted against the proposed dimensionless number ${{{{\mathscr{W}}}}}_{\theta }$ in Figure 3(b) for all considered volumes, ${{{\mathscr{S}}}}$ and *l*_*σ*_ values. The collapse of data in this plot indicates that ${{{{\mathscr{W}}}}}_{\theta }$ accurately represents the macroscopic contact angle dependence on sessile drop volume and material properties of the system, and it is actually able to characterize it.

**Fig. 4:**
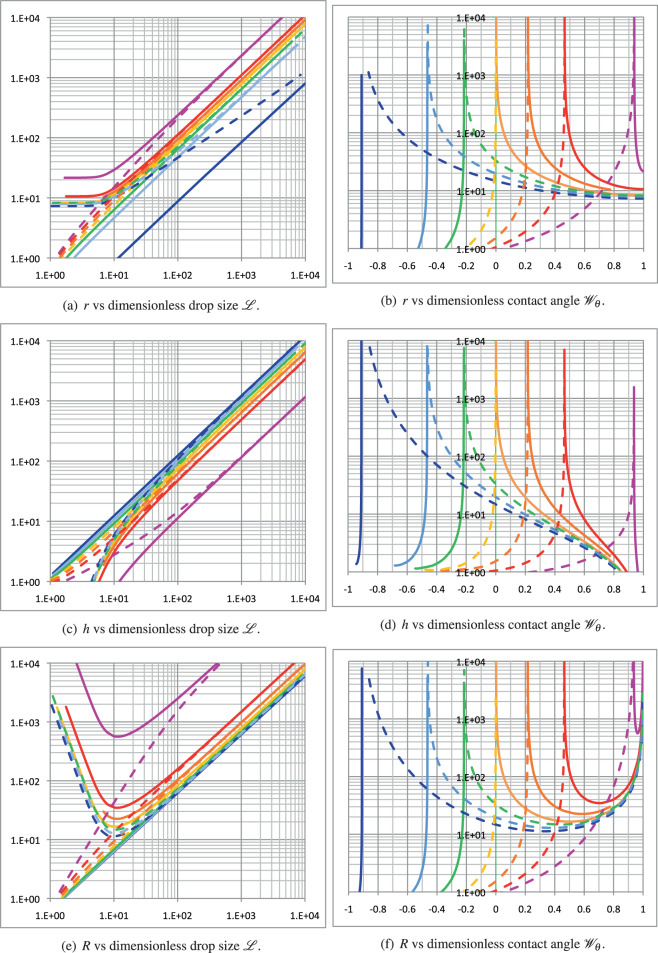
Plots of related sessile drop dimensionless quantities. **a**, **b** Wetted radius, **c**, **d** drop height, **e**, **f** sphere radius, for ${{{\mathscr{S}}}}=-0.99$ (blue), −2/3 (cyan), −1/3 (green), 0 (yellow), 1/3 (orange), 2/3 (red), and 0.99 (magenta). The solid (dashed) lines represent the plots for positive (negative) ${{{\mathscr{S}}}}/{l}_{\sigma }$.

The plots in Figure 4 display how the various dimensionless geometrical quantities related to the spherical cap model (wetted radius, height, and spherical cap radius) evolve both with respect to the dimensionless drop size and to the proposed dimensionless number ${{{{\mathscr{W}}}}}_{\theta }$. The dimensionless sessile drop radius (wetted radius, *r*) is plotted against the dimensionless drop size ${{{\mathscr{L}}}}$ in Fig. 4a and against the dimensionless contact angle ${{{{\mathscr{W}}}}}_{\theta }$ in Fig. 4b. For positive *l*_*σ*_, contact line tension induces wetting; thus, it starts with an asymptotic value that is approximately one order of magnitude greater than the smallest drop size. Its initial slope is horizontal with respect to both the dimensionless drop size and the contact angle. Then, it evolves in the transition region in a quasi-logarithmic manner and reaches the region of its asymptotic linear behavior. The higher the value of ${{{\mathscr{S}}}}$, the higher the wetted radius at a given drop size. In contrast, for negative *l*_*σ*_, the dimensionless drop radius evolves linearly with drop size above the transition region. The plot of sessile drop radius against the dimensionless contact angle ${{{{\mathscr{W}}}}}_{\theta }$ expands this underlying behavior, as shown in Fig. 4b.

The dimensionless height of the sessile drop (*h*) is plotted against the dimensionless drop size ${{{\mathscr{L}}}}$ in Fig. 4c and dimensionless contact angle ${{{{\mathscr{W}}}}}_{\theta }$ in Fig. 4d. The height clearly exhibits symmetric behavior compared with the drop wetted radius with respect to the sign of the contact line tension *l*_*σ*_. Indeed, positive values of the latter induce wetting, which translates into greater spreading and consequently a smaller drop height at a given drop size. Thus, the higher the ${{{\mathscr{S}}}}/{l}_{\sigma }$ ratio, the smaller the sessile drop height for a given sessile drop size.

Finally, the dimensionless sphere radius (*R*) is another geometric quantity of interest, as it determines the sessile drop bulk pressure for a given liquid–gas surface tension, as per the Young–Laplace equation. It is plotted against the dimensionless drop size ${{{\mathscr{L}}}}$ in Fig. 4e and dimensionless contact angle ${{{{\mathscr{W}}}}}_{\theta }$ in Fig. 4f. Unlike the dimensionless drop radius and height, the dimensionless sphere radius exhibits a peculiar behavior for positive *l*_*σ*_. It exhibits a minimum value that increases with ${{{\mathscr{S}}}}$. Therefore, since the capillary pressure evolves as 1/*R*, this minimum value is associated with an extreme bulk pressure in the sessile drop, which is of practical interest to design weightless experiments. Conversely, for negative *l*_*σ*_, the sphere radius continuously increases with decreasing drop size.

**Fig. 5:**
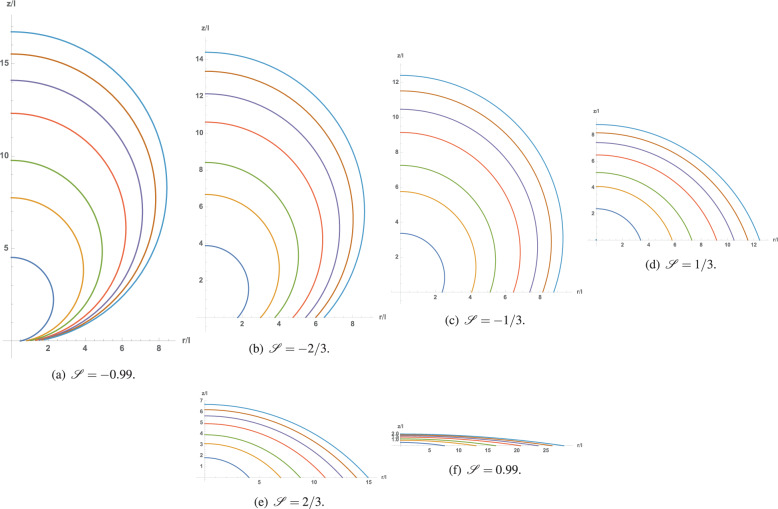
Influence of sessile drop volume on its shape in the small to medium range. ${\mathcal{V}}=5$ (dark blue), 25 (brown), 50 (purple), 100 (orange), 150 (green), 200 (yellow) and 250 (light blue), for six values of ${\mathcal{S}}$ and negative ${\mathcal{S}/l_{\sigma}}$. **a** ${\mathcal{S}}=-0.99$; (**b**) ${\mathcal{S}}=-2/3$; (**c**) ${\mathcal{S}}=-1/3$; (**d**) ${\mathcal{S}}=1/3$; (**e**) ${\mathcal{S}}=2/3$; (**f**) ${\mathcal{S}}=0.99$.

Some sessile drop shapes are shown in Fig. 5 in the axisymmetric (*r*, *z*) frame for several sample volumes, six values of ${{{\mathscr{S}}}}$, and a negative ${{{\mathscr{S}}}}/{l}_{\sigma }$ ratio. These shapes clearly display a continuous evolution of the static macroscopic contact angle with respect to ${{{\mathscr{S}}}}$ and sessile drop volume. The latter is small (few degrees) owing to the limited volume range that can be distinctively represented in the same figure; however, it is perceptible.

#### Influence of material properties for given drop volume

**Fig. 6:**
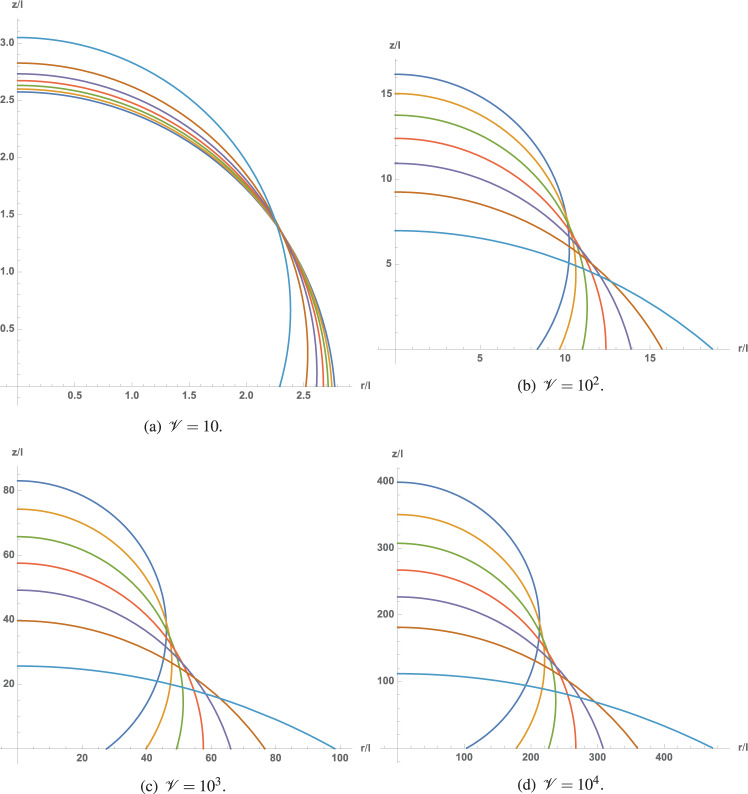
Influence of ${\mathcal{S}}$ on sessile drop shape for four volumes and seven values of ${\mathcal{S}}$, ranging from −0.99 (dark blue) to 0.99 (light blue), separated by six equidistant steps. **a** ${\mathcal{V}}=10$; (**b**) ${\mathcal{V}}\,=\,10^2$; (**c**) ${\mathcal{V}}\,=\,10^3$; (**d**) ${\mathcal{V}}\,=\,10^4$.

The sessile drop shapes of four given volumes (${{{\mathscr{V}}}}=10$, 10^2^, 10^3^, and 10^4^) are plotted in Fig. 6 for seven values of ${{{\mathscr{S}}}}$ (in the range ${{{\mathscr{S}}}}\in [-0.99,0.99]$, with six uniform increments). For the considered drop volumes, which are greater than those for which line tension dominates alone, the influence of volume on the macroscopic contact angle is stronger for negative values of ${{{\mathscr{S}}}}$ than for positive ones. Therefore, in this case, the higher the sessile drop volume, the stronger the influence of ${{{\mathscr{S}}}}$ on its shape.

## Discussion

A new macroscopic mechanical model was derived to compute the shape of weightless sessile drops in static equilibrium. Our initial motivation was to understand more clearly the repeatability issues encountered while creating sessile drops in weightlessness by injecting liquid through a small hole in a substrate^21,22^. The derived model is based on the classical Young–Laplace equation, Eq. (4), supplemented with a constitutive relationship for the pressure difference across the liquid–gas interface, Eq. (5). Assuming a flat, smooth, and homogeneous solid substrate, the model considers both the surface densities of the free energy at the solid–liquid and liquid–gas interfaces, along with the line density of energy along the contact line. The ± sign in Eq. (5) accounts for the contact angle hysteresis associated with an advancing or receding contact line. First, the resulting governing equation, Eq. (7), clearly indicates that the volume affects the macroscopic contact angle only when (*σ*_*sg*_ − *σ*_*sl*_)/*σ*_*slg*_, the ratio of the difference in the substrate surface tensions to the contact line one, is commensurable on the macroscopic scale.

The volume dependence of the macroscopic contact angle results from the solution of the derived governing equation, Eq. (7). Indeed, unlike in Boruvka and Neumann’s line tension model^4,6–8^, no a priori explicit dependence of the macroscopic contact angle on the drop radius was introduced in the proposed model. Therefore, the main outcome of the present mechanistic macroscopic model is that neither the classical Young’s equation^1^ nor the modified Young’s equation proposed by Boruvka and Neumann^8^ are appropriate for determining the macroscopic contact angle of weightless sessile drops. The evidence of this assertion is contained in Eq. (2), which is the governing equation for the macroscopic contact angle derived from our weightless model with respect to the drop wetted radius *r* (instead of drop volume *V*, that results in Eq. (7)). It reads:

2 $$(2-3\cos \theta +{\cos }^{3}\theta )+{\sin }^{2}\theta \left({{{\mathscr{S}}}}+\frac{2}{r}\frac{{\sigma }_{slg}}{{\sigma }_{lg}}-1-{\tan }^{2}\left(\frac{\theta }{2}\right)\right)=0$$

Interestingly, this governing equation expressed in drop wetted radius, Eq. (2), evidence that in weightlessness the macroscopic contact angle does not evolve as simply as in the 1/*r* Boruvka and Neumann’s model, but in much more subtle highly nonlinear way. Indeed, upon approaching the very small drop volumes for which line tension dominates, the obtained behavior significantly deviates from the 1/*r* slope because of the highly non-linear trigonometric functions involved in the spherical cap model. These latter dominate the sessile drop shape at the lower limits of macroscopic volumes, as depicted in Figs. 2 and 3a.

Furthermore, the proposed model reproduces the influence of volume on the macroscopic contact angle of a sessile drop, which has been reported in several ground experimental studies^2,3,6^. Interestingly, in weightlessness, the influence of volume on the macroscopic contact angle exists over a much wider range of drop size, as no capillary length exists, unlike under Earth’s gravity conditions. Indeed, unlike in the latter, it is not obscured by any hydrostatic pressure effect on the local interface curvature, as no capillary length exists in weightlessness. This enabled us to perform extended parametric studies on volume size effects. The proposed macroscopic model has a lower bound volume at which only line tension dictates the limit behavior, hydrophobicity (hydrophilicity), depending on whether its sign is negative (positive). Indeed, when the drop size reaches the lowest macroscopic limit, the related macroscopic contact angle approaches its limit value, ${\theta }_{lim}=\frac{\pi }{2}[1-\,{{\mbox{sign}}}\,({\sigma }_{slg})]$, regardless of the sign of ${{{\mathscr{S}}}}$. No lower sessile drop volume can be considered, as the present macroscopic model reaches its limit of physical representativity. At this lower limit, the driving mechanisms that act at microscopic scales, such as meniscus curvature, are not accounted for. Upon increasing the drop volume from this lower bound, the macroscopic contact angle continuously evolves with respect to the drop volume toward its asymptotic infinite value, ${\theta }_{\infty }=\arccos {{{\mathscr{S}}}}$. Notably, this infinite limit value coincides exactly with that from the classical Young’s equation, Eq. (1), although the latter is not involved in the present model. Therefore, the volume dependence of the macroscopic contact angle is maximum for negative values of (*σ*_*sg*_ − *σ*_*sl*_)/*σ*_*slg*_ and increases with the magnitude of $\left|{{{\mathscr{S}}}}\right|$. Furthermore, a crossover between hydrophobic (non-wetting) and hydrophilic (wetting) behaviors or vice versa always occurs for a specific sessile drop volume, ${{{{\mathscr{V}}}}}_{co}=1/{\left|{{{\mathscr{S}}}}\right|}^{3}$, when (*σ*_*sg*_ − *σ*_*sl*_)/*σ*_*slg*_ is negative, i.e., when the line tension and surface tension act in opposite directions.

Furthermore, the macroscopic contact angle can vary at static equilibrium over a broad volume range and it can increase or decrease against volume depending on the sign of the surface to line ratio of free energies until it reaches its asymptotic value. Finally, the injection pressure is not a relevant control parameter for creating in a well-controlled way sessile drops of a target volume by injecting liquid through a small hole in the substrate. Indeed, the injection pressure evolves in a strongly non-linear manner with sessile drop volume: it continuously decreases against drop volume for negative ${{{\mathscr{S}}}}/{l}_{\sigma }$ cases, whereas conversely, it first increases and then decreases (please refer to Fig. 4e). Thus, feedback control systems based on pressure only are likely to be unstable, so the authors recommend enslaving the injection mass flow rate to drop volume instead of drop pressure. Actually, a constant injection pressure would result in a continuously accelerating injection flow rate, which makes inertia terms to become more and more prevalent, and prevent to achieve at the end of the injection stage a sessile drop of prescribed target volume. Moreover, since it exists some macroscopic contact angle hysteresis from advancing and receding contact line modes, getting back from an oversized drop to an accurate target volume becomes even much trickier.

Future research must focus on experiments in weightlessness on sessile drops for much larger volume ranges than those that exist under Earth’s gravity conditions. Indeed, the present weightless model clearly indicates that the macroscopic contact angle asymptotically converges towards the value of the Young’s equation in the limit of large volumes. So, in this limit weightless experiments can provide reference data for the surface tension values (*σ*_*sg*_, *σ*_*ls*_, and *σ*_*lg*_), which are crucial for accurate predictive modeling. Then, considering on the other hand the lower volume range, one can evaluate in turn the line tension value *σ*_*slg*_, enabling us to assess the validity of the volume dependence of the macroscopic contact angle. Finally, one can check the model predictions on limit volumes for both hydrophilic and hydrophobic cases. If such experiments confirm the predictions of the proposed model, they will potentially have an impact on the current physical understanding of sessile drops, even under Earth gravity conditions. If the sessile drop volume influences the macroscopic contact angle in weightlessness, it should also have the same effect under Earth gravity. However, this is somehow blurred or screened by the self-adaption of the local interface curvature to the hydrostatic pressure, which becomes the dominant contact line mechanism as soon as the drop size exceeds some fraction of its capillary length. Therefore, the authors believe that the present weightless model could nevertheless predict some representative results either in microgravity or Earth gravity conditions, provided the relative perturbation induced by the hydrostatic pressure with respect to capillary one does not exceed few percent (below 10%), according to a first order perturbation technique. Hence, the validity of the classical Young’s equation^1^ for the macroscopic contact angle is affected to some extent by the volume dependence previously reported in ground experiments^2,4–7^.

## Methods

### A new model

Let us consider the static mechanical equilibrium of a weightless sessile drop of a given volume and with a fixed set of properties for the liquid, substrate, and surrounding gas. To prevent unnecessary modeling complexity and related issues, the solid substrate is assumed to be flat, smooth, and homogeneous. To create this sessile drop by injecting liquid through a small hole in the substrate (monotonously advancing contact line), the supplied injection pressure should overcome the free energies associated with the curved liquid–gas interface, wetting a certain amount of substrate area at the liquid–solid interface, and the advancing contact line, respectively.

#### Governing equations

Assuming a sufficiently slow injection flow rate, such that inertia and viscous dissipation terms are negligible with respect to the surface and line free energies, the mechanical work associated with liquid injection for a sessile drop of volume *V* is:

3 $${\int}_{V}\left[{p}_{i}(v)-{p}_{e}\right]dv={\int}_{{A}_{lg}}{\sigma }_{lg}\ da+{\int}_{{A}_{sl}}({\sigma }_{sl}-{\sigma }_{sg})\ da+{\int}_{{L}_{slg}}{\sigma }_{slg}\ dl$$

where *p*_*i*_ and *p*_*e*_ are the liquid drop bulk pressure and surrounding gas pressure, respectively; *σ*_*lg*_, *σ*_*sg*_, and *σ*_*sl*_ are the liquid–gas, solid–gas, and liquid–solid surface densities of the surface free energy, respectively; and *A*_*lg*_ and *A*_*sl*_ are the liquid–gas and solid–liquid interface areas, respectively. Finally, *σ*_*slg*_ is the line density of the three-phase zone free energy associated with the macroscopic contact line, defined as the perimeter of the wetted surface, *L*_*slg*_.

To proceed in this weightless static mechanical equilibrium, it is noteworthy that the bulk pressure in the liquid drop and surrounding gas pressure are both constant, so their difference behaves accordingly; this results in a constant curvature of the liquid–gas interface. Consequently, the weightless sessile drops are spherical caps. This geometrical feature and its associated trigonometric relationships are crucial for deriving any analytical expression that relates the mechanical equilibrium to quantities associated with the macroscopic shape of the sessile drop in a closed form: volume (*V*), wetted radius (*r*), height (*h*), and static macroscopic contact angle (*θ*).

A static equilibrium results in a normal and tangential macroscopic force balance at the solid–liquid and liquid–gas interfaces, respectively. Its normal component to the substrate leads to the Young–Laplace equation:

4 $${p}_{i}-{p}_{e}=2\ {\sigma }_{lg}\frac{\sin \theta }{r}=\frac{2{\sigma }_{lg}}{R}$$

where *R* is the radius of the resulting spherical cap.

To derive a well-posed governing equation, a constitutive relationship was introduced for the bulk pressure of weightless sessile drop. Therefore, assume that any difference in chemical potential between the liquid, solid substrate, and surrounding gas produces adhesive or repulsive forces at the molecular scale acting along their respective solid–liquid, liquid–gas, and three-phase zone. Then, these forces can translate into pressure in the bulk of the liquid drop. Assuming thermodynamic equilibrium, both the surface and line densities of free energy become constant, enabling the formal integration of Eq. (3). Then, dividing the integrated equation by the consistent drop volume leads to the proposed local constitutive relationship for the bulk pressure:

5 $${p}_{i}-{p}_{e}=\frac{{\sigma }_{lg}\ \pi ({r}^{2}+{h}^{2})+\left({\sigma }_{sl}-{\sigma }_{sg}\right)\pi {r}^{2}\pm {\sigma }_{slg}\ 2\pi r}{{V}_{ci}}$$

where the ± in front of the contact line free energy (*σ*_*slg*_) is associated with the contact angle hysteresis, with a positive (negative) sign for an advancing (receding) contact line. Moreover, the consistent volume integration is such that the final governing equation satisfies the surface-to-volume ratio for any spherical cap, that is, *V*_*ci*_ = 3*V*/2. Equating the bulk pressure differences from Eq. (4) and Eq. (5) and replacing *R*, *r*, and *h* with their respective trigonometric relationships defined in Eq. (6A)(6B)(6C),

6A $$R={\left[\frac{3V}{\pi (2-3\cos \theta +{\cos }^{3}\theta )}\right]}^{1/3}$$

6B $$\frac{h}{r}=\tan (\theta /2)$$

6C $$r={\left[\frac{6V}{\pi \tan (\frac{\theta }{2})[3+{\tan }^{2}(\frac{\theta }{2})]}\right]}^{1/3}$$

the following governing equation for the weightless macroscopic contact angle *θ* of the advancing contact line is finally obtained:

7 $$\begin{array}{ll}&\left[{\left(2-3\cos \theta +{\cos }^{3}\theta \right)}^{\frac{1}{3}}{\left(\tan \left(\frac{\theta }{2}\right)\left[3+{\tan }^{2}\left(\frac{\theta }{2}\right)\right]\right)}^{\frac{2}{3}}-{2}^{\frac{2}{3}}\left(1+{\tan }^{2}\left(\frac{\theta }{2}\right)-{{{\mathscr{S}}}}\right)\right]{{{{\mathscr{V}}}}}^{\frac{1}{3}}\\ &+{\left(\tan \left(\frac{\theta }{2}\right)\left[3+{\tan }^{2}\left(\frac{\theta }{2}\right)\right]\right)}^{\frac{1}{3}}=0\end{array}$$

${{{\mathscr{S}}}}$ and ${{{\mathscr{V}}}}$ are two dimensionless numbers that determine the macroscopic contact angle in weightlessness. They are related to the physical parameters of the problem, as follows:

8A $${{{\mathscr{S}}}}=\frac{{\sigma }_{sg}-{\sigma }_{sl}}{{\sigma }_{lg}}$$

8B $${{{\mathscr{V}}}}=\frac{V}{{l}_{ref}^{3}}$$

8C $${l}_{ref}={\left(\frac{16\pi }{3}\right)}^{\frac{1}{3}}\left|{l}_{\sigma }\right|$$

where ${l}_{\sigma }=\frac{{\sigma }_{slg}}{{\sigma }_{lg}}$ and *l*_*ref*_ are the physical reference lengths of the weightless sessile drop. However, another scale associated with the volume of the derived macroscopic model can also be defined:

9 $${l}_{mac}=\mathop{{{{\rm{lim}}}}}\limits_{\theta \to {\theta }_{lim}}{V}^{\frac{1}{3}},\,{{\mbox{with}}}\,{\theta }_{lim}={0}^{+}\,{{\mbox{if}}}\,{\sigma }_{slg} \, > \, 0,{{\mbox{otherwise}}}\,{\theta }_{lim}={\pi }^{-}$$

According to this definition, the minimal macroscopic length *l*_*mac*_ is the size of the weightless sessile drop above which a macroscopic contact angle can be determined using Eq. (7). Conversely, below *l*_*mac*_, we expect the presented macroscopic model to be irrelevant.

The expression of Eq. (7) is dimensionless, which reveals the direct influence of the ${{{\mathscr{S}}}}{{{{\mathscr{V}}}}}^{\frac{1}{3}}$ product on the macroscopic contact angle. Therefore, this is a key component in the definition of a dimensionless number, which quantifies the static macroscopic contact angle of a weightless sessile drop. The first method introduced herein is defined as follows:

10A $${{{{\mathscr{W}}}}}_{\theta }=\left[\frac{2}{\pi }\arccos {{{\mathscr{S}}}}+\,{{\mbox{sign}}}\,({\sigma }_{slg})-1\right]\left[(1-s){f}_{co}+s\ {f}_{pa}\right]+\,{{\mbox{sign}}}\,({\sigma }_{slg})$$

10B $$s=\left[1+\tanh \left(a{{{\mathscr{L}}}}+b\right)\right]/2$$

10C $${f}_{co}=\exp \left(-{\left[\frac{{{{\mathscr{L}}}}-1}{c}\right]}^{d}\right)$$

10D $${f}_{pa}=\frac{e}{f{{{\mathscr{L}}}}+g}+\frac{1}{{{{\mathscr{L}}}}}$$

10E $${{{\mathscr{L}}}}={V}^{\frac{1}{3}}/{l}_{mac}$$

The proposed definition of ${{{{\mathscr{W}}}}}_{\theta }$, Eq.(10A), is tailored to lie within $\left[-1,1\right]$ and to indicate the hydrophobic, neutral, or hydrophilic behavior of the weightless sessile drop for negative, null, or positive values, respectively. Moreover, it represents the signed dimensionless distance to cross-over behavior. To meet all these requirements, we introduce a sigmoid function, defined as *s* in Eq. (10B), which is built on the hyperbolic tangent for generating a smooth transition between the contrasting behaviors encountered at the limit angle and away from it. ${{{\mathscr{L}}}}$ is the dimensionless drop length for characterizing the macroscopic contact angle in weightlessness. *f*_*co*_ and *f*_*pa*_, account for the specific behaviors encountered over the entire variation range. *f*_*co*_ connects to the limit angle *θ*_*lim*_ at ${{{\mathscr{L}}}}=1$, and *f*_*pa*_ enables the transition from this connecting region to the asymptotic region of hyperbolic $1/{{{\mathscr{L}}}}$ behavior. All constants (*a*, *b*, *c*, *d*, *e*, *f*, and *g*) depend on ${{{\mathscr{S}}}}$, *l*_*σ*_, and sign(*σ*_*slg*_), which are the physical parameters of the problem.

#### Model validation using published experimental data

**Fig. 7:**
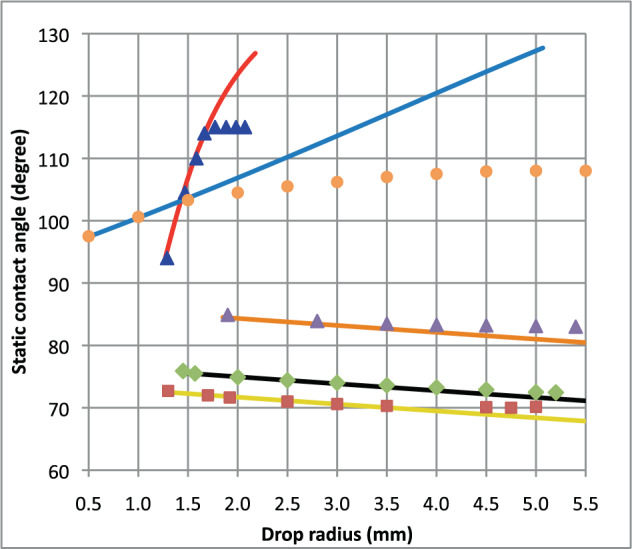
Comparison of proposed model with published experimental data. Legend: blue triangles—Herzberg and Marian^2^ (Table 1), orange solid line—present model (${{{\mathscr{S}}}}=-0.96$ and *l*_*σ*_ = 0.25 mm); orange dots—Ponter and Yekta-Fard^3^ (Figure 4), blue solid line—present model (${{{\mathscr{S}}}}=-0.77$ and *l*_*σ*_ = 0.035 mm); purple triangles—Li and Neumann^6^ (Figure 3, lower plot), orange solid line—present model (${{{\mathscr{S}}}}=0.26$ and *l*_*σ*_ = − 0.015 mm); green diamonds—Li and Neumann^6^ (Figure 3, middle plot), black solid line—present model (${{{\mathscr{S}}}}=0.42$ and *l*_*σ*_ = − 0.015 mm); red squares—Li and Neumann^6^ (Figure 3, top plot), yellow solid line—present model (${{{\mathscr{S}}}}=0.47$ and *l*_*σ*_ = −0.015 mm).

Before discussing the proposed model, it is important to validate it using existing experimental results. Unfortunately, to the best of the authors’ knowledge, none of the published experimental results on weightlessness include any quantifiable dependence of the macroscopic contact angle on volume. Therefore, the results obtained using the proposed model are plotted in Figure 7 against the experimental data obtained considering Earth’s gravity conditions.

Although a broad range of fluids, substrates, and surrounding gases are involved in the five selected experimental results^2,3,6^, fitting the ${{{\mathscr{S}}}}$ and *l*_*σ*_ parameters of the proposed model to the lower drop size experimental points in each of the considered cases enables us to determine the influence of volume on the macroscopic contact angle satisfactorily. Interestingly, the results of the proposed weightlessness model deviate from those of Earth gravity experiments as soon as the drop size reaches some fraction of its capillary length (water/air fluids: *λ*_*c*_ ≈ 2.6 mm, ethylene glycol/air fluids: *λ*_*c*_ ≈ 2.0 mm, respectively). This occurs because the influence of volume no longer exists under gravity conditions, whereas it persists in weightlessness. A sharp departure is observed for the most hydrophobic case of water on a polyethylene substrate^2^ for a drop radius exceeding *r* = 1.7 mm (drop volume from Table 1 in Herzberg and Marian^2^ translated into drop radius, as shown in Eq. (6C)). Moreover, this case is that of highest slope among the five considered cases (${{{\mathscr{S}}}}=-0.96$). For the second hydrophobic case of water on polytetrafluorethylene (PTFE), a milder deviation occurs between the present model and experimental results from Ponter and Yekta-Fard^3^ above a comparable radius of *r* > 1.7 mm. For both hydrophobic cases considered here, fairly consistent *l*_*σ*_ have been introduced to fit experimental results from Herzberg and Marian^2^ and Ponter and Yekta-Fard^3^, *l*_*σ*_ = 0.25 mm and 0.035 mm, respectively. On the other hand, for the three hydrophilic cases, either for Dodecane on FC-721 substrate and Zonyl FSC one, or ethylene glycol on DDOAB substrate^6^, only a slight volume dependence of the macroscopic contact angle is observed, which results in a minute slope for the three cases. This explains why only minor deviations can be observed for drop radii beyond *r* = 4 *mm*, cf. Fig. 7. Moreover, very small *l*_*σ*_ have been introduced to fit the experimental values from^6^ comparatively to those introduced for the two hydrophobic cases, a same *l*_*σ*_ = −0.015 mm in the three cases.

### Reporting summary

Further information on research design is available in the Nature Research Reporting Summary linked to this article.

## Supplementary information

Reporting summary

## Data Availability

The data that support the findings of this study are available from 10.5281/zenodo.4382265.
